# An Epidemiological Study of Brucellosis in Different Animal Species from the Al-Qassim Region, Saudi Arabia

**DOI:** 10.3390/vaccines11030694

**Published:** 2023-03-17

**Authors:** Abdulaziz M. Almuzaini

**Affiliations:** Department of Veterinary Medicine, College of Agriculture and Veterinary Medicine, Qassim University, Buraydah 52571, Saudi Arabia; ammzieny@qu.edu.sa

**Keywords:** brucellosis, CFT, ELISA, prevalence, RBT, zoonosis, public health, Saudi Arabia

## Abstract

Brucellosis is a zoonotic bacterial illness that affects humans and a variety of domestic animals, especially ruminants. It is mostly transmitted through the consumption of contaminated drinks, foods, undercooked meat, or unpasteurized milk or contact with infected animals. Therefore, the present study aimed to investigate the seroprevalence of brucellosis in camels, sheep, and goat herds in the Qassim region, Saudi Arabia, using commonly used diagnostic serological procedures such as the Rose Bengal test (RBT), complement fixation test (CFT), and enzyme-linked immunosorbent assay (ELISA). The seroprevalence of brucellosis in camels, sheep, and goats was determined in the selected areas using a cross-sectional study design and a total of 690 farm animals of both sexes of different ages from the three animal species (274 camels, 227 sheep, and 189 goats). According to RBT results, 65 sera were positive for brucellosis, including 15 (5.47%) for camels, 32 (14.09%) for sheep, and 18 (9.50%) for goats. CFT and c-ELISA were performed as confirmatory tests on positive samples resulting from RBT. With c-ELISA, 60 serum samples were confirmed positive, in 14 (5.10%), 30 (13.21%), and 16 (8.46%) camels, sheep, and goats, respectively. There were 59 serum samples confirmed as positive for CFT, including 14 (5.11%), 29 (12.77%), and 16 (8.46%) for camels, sheep, and goats, respectively. Overall, the highest seroprevalence of brucellosis was found in sheep while the least was found in camels from the three tests (RBT, c-ELISA, and CFT). The highest seroprevalence of brucellosis was found in sheep while the least seroprevalence was found in camels. There was also a higher seroprevalence of brucellosis among female animals than males as well as among old animals than young animals. The study, thus, demonstrates brucellosis seroprevalence among farm animals (camels, sheep, and goats) and the significance of intervention measures against brucellosis incidence in both humans and animals through the creation of public awareness and other relevant policy measures such as livestock vaccination, effective hygiene management, and adequate quarantine or serological analysis for newly introduced animals.

## 1. Introduction

A highly contagious zoonotic disease known as brucellosis is caused by the highly contagious bacteria *Brucella* spp., a Gram-negative bacterium that can be transmitted to both humans and animals and poses a significant risk to public health [1]. There is a general consensus that cattle are primarily affected by *Brucella abortus* (*B. abortus*) and less frequently by *Brucella suis* (*B. suis*) and *Brucella melitensis* (*B. melitensis*), whereas sheep and goats are most commonly affected by *B. melitensis* [2,3]. However, pigs and dogs are affected by *B. suis* and *B. canis*, respectively. Both *B. melitensis* and *B. abortus* have been mostly implicated in humans, thus, rendering cattle, sheep, and goats related to human infection [4]. Camels, on the other hand, are susceptible to both *B. melitensis* and *B. abortus* infections though they are not the primary hosts of *Brucella* [5,6]. They also exhibit only a few clinical signs in contrast to cattle, thus, making the diagnosis of camel brucellosis a bit difficult and challenging [7]. Nevertheless, there is a high chance of cross-transmission among camels, cattle, goats, sheep, and other species [8]. Recently, additional *Brucella* species such as *B. ovis or B. neotomae* (same strain as *B. canis*), *B. microti* from the common vole, *B. penibedalis* and *B. cetacia* from marine mammals, *B. inopinata* from the female breast, *B. papioni* from baboons, and *B. vulpeuis* from red foxes have been reported [9,10]. *B. melitensis*, however, is still the leading cause of human brucellosis (94.00% of cases); *B. abortus* is significantly less common (6.00%), which supports the idea that it is the most important human pathogen worldwide [11,12,13].

It is known that *Brucella* is not host specific, but it does exhibit a host preference, and spillover can occur when many host species are maintained together or when high-quality grazing areas and water supplies are shared [14]. There are a number of ways for this disease to spread to humans, including consuming unpasteurized dairy products, consuming infected tissues, and becoming in contact with sick people and animals [15,16,17,18]. People are still uncertain of the probability of passing brucellosis from person to person, but it is highly likely to happen. It has been reported in Royal Oak, Michigan, in the United States, where the bacteria were found in the wife of a microbiologist who had been infected, implying that sexual contact might be responsible for the infection [19]. Several *Brucella* species have been identified in central Saudi Arabia [20] as a result of human investigations conducted in the desert climate such as *B. melitensis* [21,22,23,24,25] and *B. abortus* [21,23,24,25]. The most common modes of transmission of brucellosis include vertical and horizontal transmissions in animals such as through abraded skin, accidental injection, inhalation of aerosolized bacteria, or ingestion of organisms, and in humans through the consumption of contaminated foods, undercooked meat, or unpasteurized milk [26,27,28,29,30]. Movement of livestock from one geographical region to another, extensive grazing, free grazing, and large herd sizes with infection also contribute to tconhe transmission of brucellosis [4]. Infected animals shed high numbers of organisms in aborted fetuses, blood, urine, milk, delivery/vaginal discharges, placentas, semen, and tissue [31].

Over half a million human cases of brucellosis are reported annually throughout the world, which makes it an extremely common bacterial zoonotic disease that affects both animals and humans [32]. Its prevalence varies widely from country to country in animals and humans [4] and about 2.4 billion people are at risk of the disease [33]. Brucellosis is associated with abortion, infertility, and decreased production of milk and meat, resulting in considerable economic impact on the animal industry worldwide [34]. The disease has been eradicated in many developed nations in spite of the fact that it is still prevalent in a number of regions, including the Mediterranean region [35], Africa [14,36], as well as some developed nations that lack livestock (such as sheep, goats, cattle, water buffaloes, camels, and pigs) due to low incomes, few resources, and a lack of resources [37]. There are variations in the prevalence of this disease throughout the world because most of the countries within Africa were documented to have a high incidence rate that was higher than that of any other country. A high number of cases were also reported in the Aseer region of the Kingdom of Saudi Arabia (KSA) between 2004 and 2012 [38].

From a public health perspective, brucellosis is usually considered an occupational health hazard that mainly affects livestock handlers such as butchers, farmers, laboratory staff, slaughter workers, and veterinarians [39,40,41]. The disease is also known as the travel-related infectious disease “gastric remittent fever”, “Malta fever”, “Mediterranean fever”, or “undulant fever” [42]. Brucellosis has been substantially eradicated in many industrialized countries, including New Zealand, Australia, Canada, Japan and some member countries of the European Union (Denmark, Belgium, Germany, Finland, Ireland, Luxembourg, Sweden, The Netherlands, the United Kingdom). However, in the Middle East, the Mediterranean region, Africa, parts of Asia, and Latin America, the disease is still a major public health issue [43,44]. Brucellosis also causes substantial economic losses in addition to other animal health, social, and public health impacts in most developing countries including some Mediterranean countries owing to the complexity of disease control and epidemiology of brucellosis [45,46]. Brucellosis generates a serious economic consequence for both the extensive and intensive livestock production systems in the tropical regions [47]. The World Health Organization (WHO) classified the genus *Brucella* as a risk group III pathogen [48]. Though the bacterium grows slowly, however, it readily survives and replicates within dendritic cells, epithelial cells, macrophages and placental trophoblasts [49]. 

The diagnosis of brucellosis is generally achieved either directly or indirectly via *Brucella* isolation or the detection of specific antibodies [50]. Nevertheless the most reliable method and a gold standard of diagnosis of *Brucella* is isolation and, then, identification of the bacteria [51]; its performance is, however, not only time-consuming and difficult but also poses a great infection risk to workers in the laboratory, thus, necessitating specific biosafety procedures. Therefore, the recognition of *Brucella*-specific antibodies is conventionally carried out using several serological tests such as the complement fixation test (CFT), Rose Bengal test (RBT), enzyme-linked immunosorbent assay (ELISA), buffered acidified plate antigen test, and the tube agglutination test. The ease of performance, field suitability, rapidity, and simplicity of RBT makes it very useful as a herd screening high sensitivity test even in remote places while confirmatory tests such as CFT and ELISA are utilized for *Brucella* diagnosis [7,40]. The World Organization for Animal Health (OIE) has also prescribed RBT as one of the recommended serological test methods for international animal trade [52]. Other common confirmatory tests of high specificity such as the indirect enzyme-linked immunosorbent assay (i-ELISA), competitive ELISA (c-ELISA), fluorescence polarization assay (FPA), and standard agglutination test (SAT) are also well known [52,53]. The direct demonstrations of the causal organism of brucellosis have also been reported using techniques such as animal inoculation, culture, immunofluorescent antibody, polymerase chain reaction (PCR), and staining [54]. While serological tests are more commonly used to detect brucellosis than other methods, some researchers believe that these tests may not be sensitive enough to detect the disease in its latent or early stages, when animals continue to be asymptomatic in the absence of any visible signs [55]. A PCR analysis can increase the sensitivity and specificity of detecting the infection and differentiation between *Brucella* species [55,56,57,58,59].

A number of endemic regions, including France, Israel, and the majority of Latin America, have now been identified as being able to control this illness. However, in Saudi Arabia, for the uncontrolled importation of animals with no suitable inspection, the disease is distributed and the country has become endemic [60]. Furthermore, Memish [61] indicates that the KSA imports from Africa several million heads of sheep and goats each year for sacrifice during the Hajj pilgrimage. To prevent an outbreak of brucellosis from spreading to Saudi Arabia, it may be necessary to restrict the importation of small ruminants from countries that have an active brucellosis outbreak, as well as to strictly enforce quarantine measures throughout the country [62]. Raw meat and unpasteurized dairy products, such as milk and cheese, are the main prevention measures for brucellosis infection, and personal protective measures such as wearing thick gloves, eye protection, and clothing for those who come into direct contact with animals should be promoted. Vaccinating animals against *B. abortus* and *B. melitensis* strains is one of the most effective ways to protect against this disease in the KSA, and regulation is necessary to ensure that all animals are properly immunized [1]. Researchers are still trying to find an effective and safe vaccine for human brucellosis in Saudi Arabia. They are testing different approaches to developing a vaccine, such as looking at the efficacy of different combinations of antigens, as well as testing for the presence of protective antibodies. The study’s objective is, thus, to investigate the brucellosis seroprevalence in camels, sheep, and goats’ herds in the Qassim region, Saudi Arabia, by commonly used diagnostic serological procedures.

## 2. Materials and Methods

### 2.1. Ethical Statement

Because there were no animal participants in this study, it did not involve ethical approval or written permission. Only serum previously collected from regular medical testing has been used, not primary animal samples. All of the clinical strains used in this study came from regular diagnostic tests. As a result, there was no attempt to obtain animal samples for the investigation.

### 2.2. Study Design

**Figure 1:**
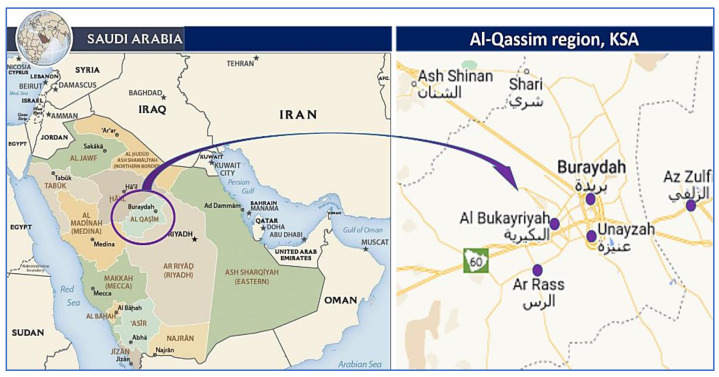
Map of Saudi Arabia with the Qassim region highlighted in purple and the study sites. Available online: maps-saudi-arabia.com/al-qassim-saudi-arabia-map (accessed on 17 February 2021).

The brucellosis seroprevalence in camels, goats, and sheep was determined using a cross-sectional study design in the selected study sites as well as the associated risk factors [2,52]. The current investigation was conducted in the north, south, east, and west of the Saudi Arabian province of Qassim (Figure 1). It is one of the thirteen provinces of Saudi Arabia and is situated in the center of the country. According to its geographical location, the area lies between the latitudes of 25°48′23′′ N and 42°52′24′′ E, and its climate is characterized by a hot summer and mild winter. A couple of distinct seasons are distinguishable in this region: one is hot from April to October and one is cold from December to March. When the hot season is in full swing from May through mid-October, there can be some extreme heat conditions. Between April and November, there is an intermediate period of warm weather. Between September 2020 and May 2022, the current cross-sectional study was conducted on camels, sheep, and goats from farms and animal hospitals within the Qassim region in order to determine the seroprevalence of brucellosis among different animal species.

### 2.3. Study Population

The methodology of the sample population was formulated from the work [40] with some modifications. Briefly, a total of 690 farm animals of both sexes from three animal species (274 camels, 227 sheep, and 189 goats) were described in Table 1. The detailed history of the animal such as age, sex, and locality were also recorded. Due to the relatively low number of male camels (one male for every four females) [63], sheep, and goats (one male for every five females) [64] in the Qassim region, the majority of serum samples in the current investigation were collected from females.

### 2.4. Sampling Technique and Collection 

By using a purposive sampling method, medium-sized, large-scale, and small-scale farms (camels, sheep, and goats) were chosen based on their proximity to each other and the number of animals they own. This method of sampling allowed us to choose a representative sample of farms of different sizes, while also ensuring that the farms were located close enough to each other to be easily accessible. The aim of using a purposive (convenient) sampling method is to use a sample that can be gathered in a simple and convenient manner, such as from a nearby herd, kennel, or from a volunteer owner. Among the herds that were included in this study, only those with a history of abortion were considered. About 5 mL of whole blood was taken from the jugular vein of each study animal (camels, sheep, and goats) and labeled with animal ID, age, location, and sex (Table 1). The sera were stored at −20 °C before serological analysis. The total number of sera samples collected from camels, sheep, and goats herds were 274, 227, and 189, respectively. Each serum sample was collected using a micropipette, which was then placed in an Eppendorf tube following the completion of the collection. To follow the recommendations of OIE [65], serum samples were stored at −20 °C until they were required to be used. This is because the OIE recommends that serum samples be stored at −20 °C in order to prevent degradation of the sample and to ensure that the results are accurate.

### 2.5. Serological Analysis of Samples

Serological tests were performed in a microbiology laboratory in accordance with WHO standards for diagnosing brucellosis in small ruminants and camels using the RBT, CFT, and A competitive enzyme-linked immunosorbent assay (c-ELISA) for diagnosis of brucellosis in small ruminants and camels. 

### 2.6. Rose Bengal Test (RBT)

The RBT was used for screening the sera samples, according to the OIE Manual [66]. The confirmatory, as well as the screening tests, were done at the Microbiology laboratory at the Public health and health informatics college of Qassim University. The antibodies to *Brucella* species were detected by RBT using a commercially available test kit (Anigen Rapid GS, *Brucella* Ab Test Kit), which was carried out following the manufacturer’s instructions. The positive sera were further retested by the c-ELISA Kit (SVANOVIR”*Brucella*-Ab, Sweden) and CFT. RBT was performed according to the procedures described by the manufacturer. The results were monitored with the standard negative and positive controls. The absence or presence, within four minutes of reaction, of a visible agglutination was considered indicative of the absence or presence of antibodies in the tested samples, respectively. All the test results were interpreted at 20 min. 

### 2.7. Competitive Enzyme-Linked Immunosorbent Assay 

A competitive ELISA test was performed on all the sera detected positive by RBT with the c-ELISA Kit (SVANOVIR”*Brucella*-Ab, Sweden), following the manufacturer’s instructions. Then, the values of the optical density (OD) were taken at 450 nm (Thermo Scientific, Waltham, MA, USA). A sample with OD ≤60% of the conjugate control wells is considered a positive sample while those >60% of the conjugate control wells are considered negative. In brief, the microplates were coated with whole-cell, heat-killed B melitensis NI cultures in phosphate buffer saline (OD_600_ about 1.0) 10 µL/well, and then incubated at 40–50 °C until the liquid evaporated entirely. After that, the antigens were immersed in a solution of 5% glutaraldehyde in 0.1 M sodium bicarbonate and allowed to fix overnight at room temperature in this solution. Each well was blocked with 5% skim milk for an hour at 37 °C, and then diluted 1:20 in blocking agents with 50 µL of serum and 1:400 in monoclonal antibody (3-F9) supernatants. During the incubation period of one hour at 37 °C, the plates were shaken for the first three minutes of the process. The glutaraldehyde solution covered the bottom of the plate, so the optical density was measured using wavelengths of 450 nm.

### 2.8. Complement Fixation Test (CFT)

The presence of anti-*Brucella* antibody was detected in the sera, and a more specific confirmatory CFT was used to further retest the sera detected positive by *RBT* following the recommended OIE procedures with the standard *B. abortus* antigen. Briefly, after heating the serum sample to approximately 56 °C, it was filtered to remove any complement proteins already present in the serum sample. In order to avoid cross-reactive anti-RBC antibodies from interfering with the test, the washed red blood cells of sheep were adsorbed with serum to prevent antibody cross-reactivity. At a temperature of 37 °C, the sample was incubated for 30 min after the addition of antigen and complement. After the indicator system had been applied to a sample, the sample was examined for any changes caused by hemolysis.

### 2.9. Statistical Analysis

All the data obtained from the three serological tests were recorded in a Microsoft Excel spreadsheet. The data were then analyzed by descriptive statistics and Pearson Correlation using the SPSS version 21.0 statistical package. The animals that tested positive for each of the RBT, c-ELISA, and CFT were defined as seropositive. The level of the individual animal seroprevalence was determined based on RBT, c-ELISA, and CFT positive results divided by the total size (number) of animals tested. The proportions (percentages) of males and females; old and young animals; as well as the geographical locations of the positive animals based on the RBT only were also estimated. The agreements between the RBT and ELISA as well as RBT and CFT results were determined using Kappa statistics. 

## 3. Results 

A total of 690 sera of farm animals of both sexes and different age groups (274 camels, 227 sheep, and 189 goats), collected from the north, south, east, and south of Qassim region, Saudi Arabia, were screened using RBT. The results showed that a total of 65 sera from 15 (5.47%), 32 (14.09%), and 18 (9.50%) camels, sheep, and goats, respectively, which were positive for the presence of brucellosis (Table 2). These were further tested with CFT and c-ELISA as confirmatory tests. The results demonstrated that only 60 out of the 65 sera were confirmed positive with c-ELISA from 14 (5.10%), 30 (13.21%), and 16 (8.46%) camels, sheep, and goats, respectively. Similarly, only 59 out of the 65 sera were confirmed positive with CFT from 14 (5.10%), 29 (12.77%), and 16 (8.46%) camels, sheep, and goats, respectively (Table 2). Overall, the highest seroprevalence of brucellosis was found in sheep while the least was found in camels from the three tests (RBT, c-ELISA, and CFT).

## 4. Correlation Studies 

The results of the regression analysis among the serological tests using Pearson correlation are shown in Table 3.

The comparison of the positive cases between the two sexes of the animals (camels, sheep, and goats) using RBT showed that the vast majority of the positive cases were from female animals. The results demonstrated that 13 (86.67%), 28 (87.50%), and 16 (88.89%) of all the positive cases from camels, sheep, and goats, respectively, were from female animals while only 2 (13.33%), 4 (12.50%), and 2 (11.11%) of all the positive cases from camels, sheep, and goats, respectively, were from male animals (Table 4).

Similarly, the comparison of the positive cases between the old and young animals (camels, sheep, and goats) using RBT showed that the vast majority of the positive cases were from old animals. The results demonstrated that 12 (80.00%), 26 (81.25%), and 15 (83.33%) of all the positive cases from camels, sheep, and goats, respectively, were from old animals while only 3 (20.00%), 6 (18.75%), and 3 (16.67%) of all the positive cases from camels, sheep, and goats, respectively, were from young animals (Table 3). The comparison of the positive cases in the selected districts (north, south, east, and west) of the Qassim region, Saudi Arabia, among the animals (camels, sheep, and goats) using RBT demonstrated that most of the positive cases were found in the Western part. However, there were positive cases of brucellosis in camels, sheep, and goats from all four zones except for sheep in the Eastern part (Table 4).

## 5. Discussion

The livelihoods of more than 1.7 billion underprivileged people globally are supported by livestock with huge demands for livestock products contributing to the growth of secondary job opportunities in addition to the main businesses of animal husbandry, transportation, slaughterhouses, and feed production [67]. Human populations greatly depend on domestic animals for the production of dairy products, fat, leather, meat, eggs, fibers, fertilizers, wool, and recreational activities. Thus, these animals play an important economic and social role for pastoralists in different parts of the world. Brucellosis, however, can affect their productivity and production. Brucellosis is regarded as one of the most lethal zoonotic diseases that affects both animals and humans. It is caused by the *Brucella* species with widely varying prevalence from one country to another both in animals and humans [42,45]. People working in the livestock industry are considered to be at risk of contracting Brucellosis because of its occupational hazards. Those who work with animals are exposed to blood, placenta, fetuses, and uterine fluids, which increases the likelihood that they will become infected. Farmers, butchers, hunters, veterinarians, epidemiologists, and laboratory workers are at the greatest risk of this type of transmission [68].

Brucellosis is responsible for a substantial economic burden to society and remains a huge threat to human mental and physical health. Previous studies indicated that community-acquired brucellosis can result in intracerebral granulomas [69] and granuloma in the Central Nervous System [70]. There are many risk factors that contribute to the development of human brucellosis, such as sociodemographic factors, transmission methods, contact with animals and their products, milking of animals, sharing water sources with animals, helping animals give birth, and ingesting animal urine [71,72]. Thus, targeting only one or a few of these factors is not sufficient to cope with the prevailing situations. Therefore, the ideal controlling and preventing strategy for brucellosis, similar to other zoonoses, is to focus on the complicated interaction between human-animal-environment interfaces. This is essential for public health interventions to alleviate disease risk [73]. Brucellosis control has become a target of economic development by the WHO and other development agencies due to the huge threat to human health and its high endemic level of animal brucellosis. The disease has not been given adequate attention in the national health system in many low-income countries despite its high economic burden [43]. 

The control of animal brucellosis largely depends on the accurate determination of its real prevalence based on the sensitivity of infected animal detection and the identification of *Brucella species* [74]. Furthermore, an effective surveillance system and control measures are required for timely updating of the true prevalence data about the diseases [75]. Therefore, its proper diagnosis remains the cornerstone for any control or eradication program. The diagnosis of brucellosis, however, always requires laboratory screening and confirmation using common bacteriological, molecular, and serological techniques [76,77]. Thus, the present study aimed to examine the brucellosis prevalence in camels, sheep, and goat herds in the Qassim region, Saudi Arabia, using RBT and was confirmed by c-ELISA and CFT. Bacteriological and immunological tests are the main diagnostic tests for brucellosis in sheep [78]. The classical RBT is used as a screening test, wherein the serum antibodies agglutinate with a stained whole cell preparation of dead *Brucella* while other tests including CFT, the Wright or serum agglutination test (SAT), and ELISA are used as confirmatory tests. The ELISA test is also able to roughly access the stage of illness and also discriminate between specific IgM and IgG antibodies’ presence [54].

The results, however, show a strong positive correlation among all the three serological tests (RBT, ELISA, and CFT) examined in this study [41,79]. Nevertheless, a more inclusive study comprising more sera might be needed to assess the performance of these serological tests in Saudi Arabia. In this study, the highest seroprevalence of brucellosis was found in sheep followed by goats while the least seroprevalence was found in camels. Some of the results are in disagreement with the literature [29,39] where it was reported that goats have a higher prevalence of brucellosis than other species. The reason for the differences might be connected to factors such as sample size, production system, breeds, species age, sex, climate, environment, and geographical locations [53,54]. 

In the current study, there is also a higher seroprevalence of brucellosis among female camels, sheep, and goats as compared to male animals. Similarly, older animals have a higher seroprevalence of brucellosis than their younger counterparts. In addition, the longer the duration of exposure, the more time the organism has to spread and reproduce, resulting in a higher concentration of the organism in the environment. This increases the chances of the animal coming into contact with the organism and becoming infected. Though it could be argued that seropositivity of brucellosis increases with advancing ages and is more prevalent in females than males, the data suggest some biases because the majority of the tested animals were old and female. The age effect could, however, be modified by sex. Nevertheless, the results are in agreement with the literature [28,54,80]. Similarly, in a meta-analysis work of [29], it was also reported that the estimated probability value suggested that post-pubertal or older animals are more likely to be seropositive than young or pre-pubertal animals due to advancing ages. The author also reported that there was a high tendency of females to be slightly more seropositive than males because females were kept longer than males and the population of females was higher than that of males in the sampling procedures. However, other studies have reported a higher seroprevalence in males than females [53], though male animals are generally at a higher risk compared to females and are also more exposed to infected females outside or within the herd/flock while searching for females in heat and under natural breeding conditions.

The current study shows very good and significant (*p* = 0.000) agreements between the RBT and ELISA as well as RBT and CFT using the Kappa Measure of Agreement. According to the literature [81], the Kappa statistic with a value of 0.8 and above denotes a very good agreement. Overall, is a worrying seroprevalence of brucellosis in camels, sheep, and goats in all the studied areas. According to the literature [27,28], seroprevalence above 5% in animals or humans is important and indicates an endemic status. Thus, adequate and effective public health education campaigns are essential for the prevention of animal brucellosis and better management of human health [48,82,83].

## 6. Conclusions

The highest seroprevalence of brucellosis was found in sheep while the least seroprevalence was found in camels. There was also a higher seroprevalence of brucellosis among female animals than males as well as among old animals than young animals. The study, thus, demonstrates the seroprevalence of brucellosis and its profile among farm animals (camels, sheep, and goats) and the need for intervention measures against brucellosis incidence in both humans and animals through and public awareness creation and other relevant policy measures such as livestock vaccination, effective hygiene management, and adequate quarantine or serological analysis for newly introduced animals. 

## Figures and Tables

**Table 1:** Details of animals’ sex, age, and locality.

Descriptions	Categories	Camels	Sheep	Goats
Sex	Male	50	32	18
	Female	224	195	171
	Total	274	227	189
Age Description	* Young	37	11	7
	* Old	237	216	182
	Total	274	227	189
Geographical descriptions	North	55	33	29
	South	68	52	64
	East	73	62	35
	West	78	80	61
	Total	274	227	189

* For camels, old ≥5 years and young ≤5 years. For sheep and goats, old ≥6 months and young ≤6 months.

**Table 2:** Detailed profile of serological immunoassays in different animal species.

Serologial Test	Camel Serum Samples					Sheep Serum Samples					Goat Serum Samples				
	North 55	South 68	East 73	West 78	Total 274	North 33	South 52	East 62	West 80	Total 227	North 29	South 64	East 35	West 61	Total 189
RBT + ve	3	5	3	4	15	6	12	5	9	32	6	3	2	7	18
c-ELISA + ve	3	7	2	2	14	4	11	6	9	30	5	3	3	5	16
CFT + ve	3	6	2	3	14	5	11	4	9	29	6	3	3	4	16

**Table 3:** Pearson’s correlation coefficients among the serological tests.

Serological Tests	RBT	c-ELISA	CFT
RBT	1.000	0.999 *	0.999 *
ELISA	0.999 *	1.000	1.000 **
CFT	0.999 *	1.000 **	1.000

Complement fixation test (CFT); Rose Bengal test (RBT); competitive enzyme-linked immunosorbent assay (c-ELISA); N = 3, * Correlation is significant at the 0.05 level (2-tailed) while ** means the correlation is significant at the 0.01 level (2-tailed).

**Table 4:** Distribution of seroprevalence of brucellosis in camels, sheep, and goats.

	Camels		Sheep		Goats	
	Number of Positive Cases	Percentage (%)	Number of Positive Cases	Percentage (%)	Number of Positive Cases	Percentage (%)
Male	2	13.33	4	12.50	2	11.11
Female	13	86.67	28	87.50	16	88.89
Total	15	100	32	100	18	100
* Young	3	20.00	6	18.75	3	16.67
* Old	12	80.00	26	81.25	15	83.33
Total	15	100	32	100	18	100
North	3	20.00	4	12.50	5	27.78
South	4	26.67	6	18.75	4	22.22
East	3	20.00	0	0.00	2	11.11
West	3	33.33	22	68.75	7	38.89
Total	15	100	32	100	18	100

* For camels, old ≥5 years and young ≤5 years. For sheep and goats, old ≥6 months and young ≤6 months.

## Data Availability

Not applicable.
